# Meteorological Register for November, 1817

**Published:** 1818-01

**Authors:** John Bailey

**Affiliations:** Surgeon, &c.


### Meteorological Register for November, 1817.

*Kept at Harwich, in Essex.*

By JOHN BAILEY, Surgeon, &c.

| Moon's Age. | | Atmospherical Pressure. Morn. | Atmospherical Pressure. Ev. | Temp. | Wind. | Remarks. | General Statement of Diseases. |
|---|---|---|---|---|---|---|---|
| | 1 | 30 1 | 30 2½ | 47 | W | Rain | |
| ☾ | 2 | 1½ | 1 | 52 | W | Cloudy | Parotidea |
| | 3 | 1½ | 1½ | 53 | SW | Fog | |
| | 4 | ½ | | 53 | S | ditto | Typhus mi- |
| | 5 | 29 9½ | 29 9 | 50 | | ditto | tior |
| | 6 | 9 | 9 | 52 | | ditto | |
| | 7 | 7½ | 5 | 53 | SE | Fine & clear | |
| | 8 | 5 | 5 | 54 | S | Rain | |
| ● | 9 | 6 | 8 | 52 | S | | |
| | 10 | 9 | 9 | 54 | S | | |
| | 11 | 7 | 7 | 52 | SW | Rain | |
| | 12 | 7 | 4 | 53 | S | Cloudy | |
| | 13 | 6½ | 6 | 51 | SW | ditto | |
| | 14 | 5 | 5 | 53 | SW | Rain | |
| ☽ | 15 | 3 | 6 | 55 | SW | ditto | |
| | 16 | 7½ | 9 | 55 | E | Fine & clear | |
| | 17 | 30 1 | 30 1 | 57 | W | Cloudy | |
| | 18 | | | 58 | W | | |
| | 19 | 3 | 3 | 48 | N | | |
| | 20 | 3 | 1 | 42 | N | Thick fog | |
| | 21 | 29 8 | 29 8 | 45 | W | Fine | |
| | 22 | 30 | 30 | 50 | | | |
| ○ | 23 | | 29 9½ | 46 | NW | | |
| | 24 | 29 7½ | 7 | 49 | NW | Rain | |
| | 25 | 9 | 9 | 41 | N | Gale of wind | |
| | 26 | 9 | 9 | 50 | W | | |
| | 27 | 30 | 30 | 49 | W | | |
| | 28 | | | 50 | W | | |
| | 29 | 29 8½ | 29 8½ | 50 | SW | | |
| | 30 | | | 54 | W | | |

The milder form of Typhus Fever has prevailed throughout the present month, attended, in some instances, with so much severity of symptoms, as to lead to the extinction of life. From the great dislike which persons, in all ranks of life, have to the use of medical remedies, and from their repugnance to abstain either from the pursuits of pleasure or of business, it is usual, upon the examination of patients labouring under this affliction, to find, that the commencement of their ailment lies far anterior to the period of its recital to their medical attendant; and he generally has to deplore the waste of that time when his exertions might have been followed, either by a removal of the impending mischief, or by a diminution of its activity. Your Reporter has had before him, this month, ample proof in support of the foregoing remark, and some reason for regret at the issue of several cases.

Parotidea, in all its degrees of violence, from simple stiffness of the jaws to extreme anguish, enlargement, and suppuration, has held its seat in this town and neighbourhood for many months. This is, for the most part, a disease to which children and young persons are subject; but we have seen it lately affecting, with great severity of attack and obstinacy of continuance, persons in the middle stage of life, occasioning not only intense local suffering, but great constitutional disorder, and leaving behind it, in the general debility, strong marks of its influence over the system at large. So far as general rules can apply, your Reporter recommends a soluble state of the bowels, with a spare diet, and diminished temperature of the enlarged parts by evaporating lotions, as this treatment is the most likely to be found successful in these cases.

